# Chemprop: A Machine Learning Package for Chemical Property Prediction

**DOI:** 10.1021/acs.jcim.3c01250

**Published:** 2023-12-26

**Authors:** Esther Heid, Kevin P. Greenman, Yunsie Chung, Shih-Cheng Li, David E. Graff, Florence H. Vermeire, Haoyang Wu, William H. Green, Charles J. McGill

**Affiliations:** † Department of Chemical Engineering, Massachusetts Institute of Technology, Cambridge, Massachusetts 02139, United States; ‡ Institute of Materials Chemistry, TU Wien, 1060 Vienna, Austria; § Department of Chemical Engineering, National Taiwan University, Taipei 10617, Taiwan; ∥ Department of Chemistry and Chemical Biology, Harvard University, Cambridge, Massachusetts 02138, United States; ⊥ Department of Chemical Engineering, KU Leuven, Celestijnenlaan 200F, B-3001 Leuven, Belgium; # Department of Chemical and Life Science Engineering, Virginia Commonwealth University, Richmond, Virginia 23284, United States

## Abstract

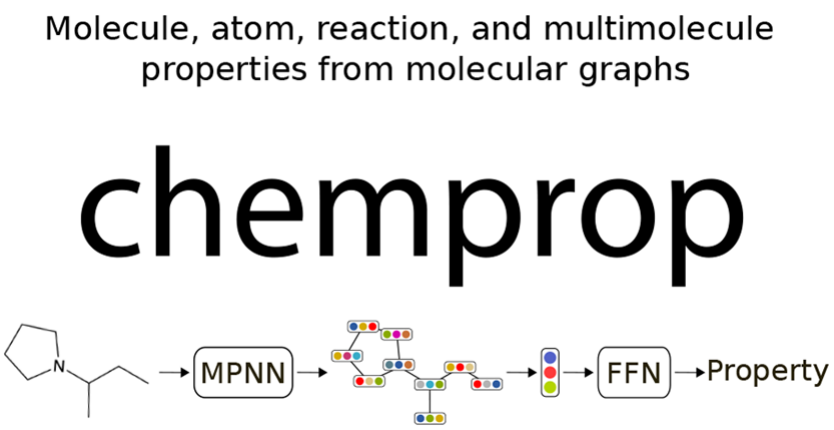

Deep learning has become a powerful and frequently employed tool for the prediction of molecular properties, thus creating a need for open-source and versatile software solutions that can be operated by nonexperts. Among the current approaches, directed message-passing neural networks (D-MPNNs) have proven to perform well on a variety of property prediction tasks. The software package Chemprop implements the D-MPNN architecture and offers simple, easy, and fast access to machine-learned molecular properties. Compared to its initial version, we present a multitude of new Chemprop functionalities such as the support of multimolecule properties, reactions, atom/bond-level properties, and spectra. Further, we incorporate various uncertainty quantification and calibration methods along with related metrics as well as pretraining and transfer learning workflows, improved hyperparameter optimization, and other customization options concerning loss functions or atom/bond features. We benchmark D-MPNN models trained using Chemprop with the new reaction, atom-level, and spectra functionality on a variety of property prediction data sets, including MoleculeNet and SAMPL, and observe state-of-the-art performance on the prediction of water-octanol partition coefficients, reaction barrier heights, atomic partial charges, and absorption spectra. Chemprop enables out-of-the-box training of D-MPNN models for a variety of problem settings in fast, user-friendly, and open-source software.

## 1 Introduction

Machine learning in general and especially deep learning has become a powerful tool in various fields of chemistry. Applications range from the prediction of physicochemical^1−9^ and pharmacological^10^ properties of molecules to the design of molecules or materials with certain properties,^11−13^ the exploration of chemical synthesis pathways,^14−27^ or the prediction of properties important for chemical analysis like IR,^28^ UV/vis,^29^ or mass spectra.^30−33^

Many combinations of molecular representations and model architectures have been developed to extract features from molecules and predict molecular properties. Molecules can be represented as graphs, strings, precomputed feature vectors, or sets of atomic coordinates and processed using graph-convolutional neural networks, transformers, or feed-forward neural networks to train predictive models. While early works focused on handmade features or simple fingerprinting methods combined with kernel regression or neural networks,^34^ the current state-of-the-art has shifted to end-to-end trainable models which directly learn to extract their own features.^35^ Here, the models can achieve extreme complexity based on the mechanisms of information exchange between parts of the molecule. For example, graph convolutional neural networks (GCNNs) extract local information from the molecular graph for single or small groups of atoms and use that information to update the immediate neighborhood.^1−3,36^ They offer robust performance for properties dependent on the local structure and if the three-dimensional conformation of a molecule is not known or not relevant for a prediction task. Graph attention transformers allow for a less local information exchange via attention layers, which learn to accumulate the features of atoms both close and far away in the graph.^37,38^ Another important line of research comprises the prediction of properties dependent on the three-dimensional conformation of a molecule, such as the prediction of properties obtained from quantum mechanics.^2,39−41^ Finally, transformer models from natural language processing can be trained on string representations such as SMILES or SELFIES, also leading to promising results.^42−45^ In this work, we discuss our application of GCNNs, namely, Chemprop,^36^ a directed-message passing algorithm derived from the seminal work of Gilmer et al.^1^

An early version of Chemprop was published in ref (36). Since then, the software has substantially evolved and now includes a vast collection of new features. For example, Chemprop is now able to predict properties for systems containing multiple molecules, such as solute/solvent combinations or reactions with and without solvent. It can train on molecular targets, spectra, or atom/bond-level targets and output the latent representation for analysis of the learned feature embedding. Available uncertainty metrics include popular approaches, such as ensembling, mean-variance estimation, and evidential learning. Chemprop is thus a general and versatile deep learning toolbox and enjoys a wide user base.

The remainder of the article is structured as follows: First, we summarize the architecture of Chemprop. We discuss a selection of Chemprop features with a focus on features introduced after the initial release of Chemprop. We then conclude the main body of the article and provide details on the data and software, which we have open-sourced including all scripts to allow for full reproducibility. Alongside the main body of this article, we provide Supporting Information that contains further model design details; descriptions of the data acquisition, preprocessing, and splitting of all data sets used in benchmarking; and the results of Chemprop benchmarks on a variety of data sets showcasing its performance on both simple and advanced prediction tasks.

## 2 Model Structure

**Figure 1:**
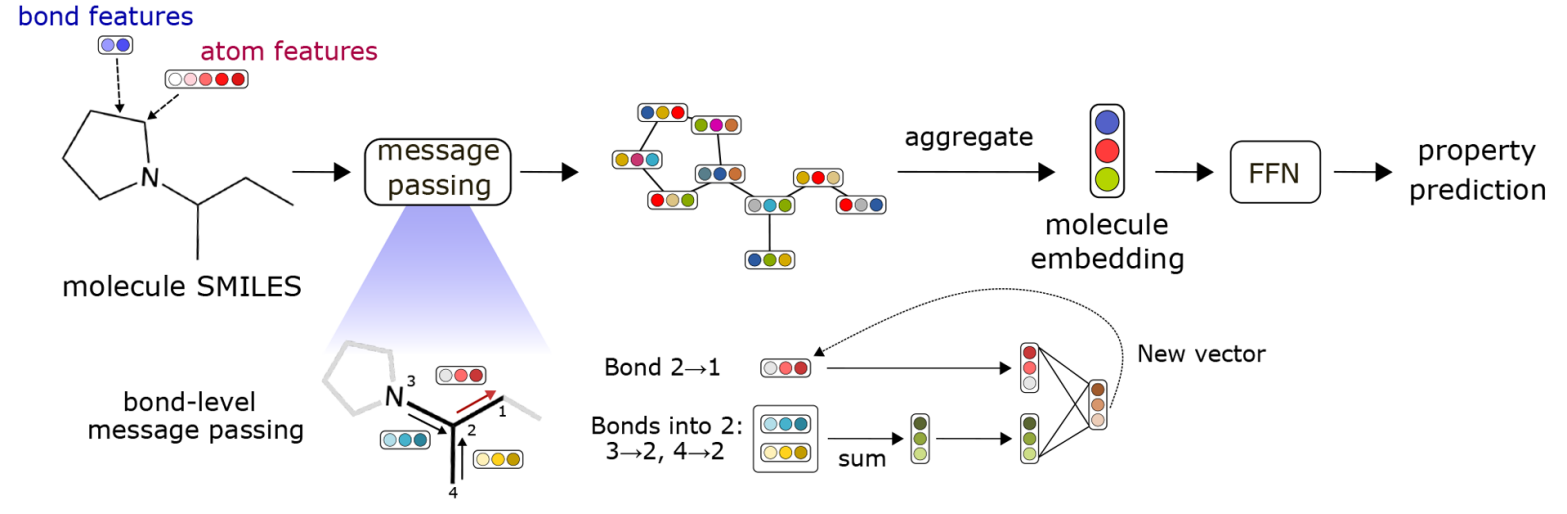
Overview of the architecture of Chemprop. The message passing update of the hidden vector for directed edge 2 → 1 is expanded for demonstration.

Chemprop consists of four modules: (1) a local features encoding function, (2) a directed message passing neural network (D-MPNN) to learn atomic embeddings from the local features, (3) an aggregation function to join atomic embeddings into molecular embeddings, and (4) a standard feed-forward neural network (FFN) for the transformation of molecular embeddings to target properties, summarized in Figure 1. The D-MPNN is a class of graph-convolutional neural networks (GCNN), which updates hidden representations of the vertices *V* and edges *E* of a graph *G* based on the local environment. In the following, we use bold lower case to denote vectors, bold upper case to denote matrices, and italic light font for scalars and objects.

For a molecule, the molecule SMILES string is used as input, which is then transformed to a molecular graph using RDKit,^46^ where atoms correspond to vertices and bonds to edges. Initial features are constructed based on the identity and topology of each atom and bond. For each vertex *v*, initial feature vectors {**x**_*v*_ |*v* ∈ *V*} are obtained from a one-hot encoding of the atomic number, number of bonds linked to each atom, formal charge, chirality (if encoded in the SMILES), number of hydrogens, hybridization, and aromaticity of the atom, as well as the atomic mass (divided by 100 for scaling). For each edge *e*, initial feature vectors {**e**_*vw*_|{*v*, *w*} ∈ *E*} arise from the bond type, whether the bond is conjugated or in a ring, and whether it contains stereochemical information, such as a cis/trans double bond. The D-MPNN uses directed edges in a graph to pass information, where each undirected edge (bond) has two corresponding directed edges, one in each direction. Initial directed edge features **e**_*vw*_^d^ are obtained via simple concatenation of the atom features of the first atom of a bond **x**_*v*_ to the respective undirected bond features **e**_*vw*_
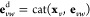
1where cat() denotes simple concatenation. The directed edges **e**_*vw*_^d^ and **e**_*wv*_^d^ are distinguished only by the choice of which atom to use in eq 1. Chemprop also offers the option to read in custom atom and bond features in addition to or as a replacement for the default features, as described in SI Section S1.2, and thus offers full control of the initial features. In summary, Module 1 of Chemprop constructs atom and directed bond feature vectors **x**_*v*_ and **e**_*vw*_^d^ from the input molecules.

The initial atom and bond features are then passed to a D-MPNN. In a D-MPNN structure, messages are passed between directed edges rather than between nodes as would be done in a traditional MPNN. To construct the hidden directed edge features **h**_*vw*_^0^ of hidden size *h*, the initial directed edge features **e**_*vw*_^d^ are passed through a single neural network layer with learnable weights 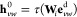
2and a nonlinear activation function τ which can be chosen by the user (default ReLU). The size *h* of **h**_*vw*_^0^ can be chosen by the user (default 300). The size of **e**_*vw*_^d^, which we term *h*_*i*_, is set by the lengths of initial feature encodings, per eq 1. The directed edge features are then iteratively updated based on the local environment via *T* (default 3) message passing steps
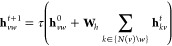
3until *t* + 1 = *T*, where  and *N*(*v*)\*w* denotes the neighbors of node *v* excluding *w*. The opposite facing directed edge is excluded from the message passing update for increased numerical stability (see Mahé et al.^47^). Finally, the updated hidden states **h**_*vw*_^*T*^ are aggregated into atomic embeddings via
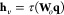
4where **q** is a concatenation of the initial atom features **x**_*v*_ and the sum of all incoming directed edge hidden states
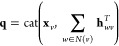
5with . Here, *h*_*o*_ is the size of **q**, i.e., the sum of the hidden size *h* and the size of **x**_*v*_. In summary, in Module 2, the D-MPNN weights **W**_*i*_, **W**_*h*_, and **W**_*o*_ are learned from the training data, outputting learnable atomic embeddings **h**_*v*_. Customizations and hyperparameter tuning include the choice of the activation function τ, the hidden size *h*, and the number of message passing steps *T*. Chemprop offers the option to add bias terms to all neural network layers (defaults to False).

The atomic embeddings **h**_*v*_ of all atoms in a molecule are then aggregated into a single molecular embedding **h**_*m*_ via
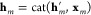
6with
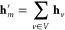
7where **x**_*m*_ is an optional vector of additional molecular features. Chemprop offers three aggregation options: summation (as shown in eq 7), a scaled sum (divided by a user specified scaler, called a norm within Chemprop), or an average (the default aggregation). Schweidtmann et al.^48^ compared the performance of such aggregation functions for different data sets. The optional additional molecular features, **x**_*m*_, may be provided features from outside sources (SI Section S2) or generated engineered fingerprints (Morgan circular fingerprints^49^ and RDKIT 2D fingerprints^46^ are implemented in Chemprop). By default, **x**_*m*_ is empty such that the molecular embedding is simply an aggregation over atomic embeddings, i.e., **h**_*m*_ = **h**_*m*_^′^ . In summary, Module 3 produces molecular embeddings **h**_*m*_ of length *h* plus the size of **x**_*m*_. Chemprop offers the option to circumvent Modules 1–3 and only using **x**_*m*_ as fixed molecular embedding, so that **h**_*m*_ = **x**_*m*_.

Finally, in the last module, molecular target properties are learned from the molecular embeddings **h**_*m*_ via a feed-forward neural network, where the number of layers (default 2) and the number of hidden neurons (default 300) can be chosen by the user. The number of input neurons is set by the length of **h**_*m*_, and the number of output neurons is set by the number of targets. The activation function between linear layers is set to be the same as in the D-MPNN, and bias is turned on per default. For binary classification tasks, the final model output is passed through a sigmoid function to constrain values to the range (0,1). For multiclass classification, the final model output is transformed with a softmax function, such that the classification scores sum to 1 across classes.

Chemprop is fully end-to-end trainable, so that the weights for D-MPNN and FFN are updated simultaneously. Users have the option to train models using cross-validation and ensembles of submodels. By default, a single model is trained on a random data split for 30 epochs. We note that small data sets need a much larger number of epochs to train and advise to check for convergence of the learning curve. Chemprop uses the Adam optimizer.^50^ The default learning rate schedule increases the learning rate linearly from 10^–4^ to 10^–3^ for the first two warmup epochs and then decreases the learning rate exponentially from 10^–3^ to 10^–4^ for the remaining epochs. By default, a batch size of 50 data points is used for each optimizer step. Early stopping and dropout are available as means of regularization. The PyTorch backend of Chemprop enables seamless GPU acceleration of both model training and inference. The acceleration of training and inference processes when used with a GPU can be significant, as shown in SI Section S3.3.

## 3 Discussion of Features

Table 1 lists a nonexhaustive selection of studies based on Chemprop, showcasing its versatility and applicability for the prediction of a large variety of chemical properties, but also its ease of use. Models can be trained and tested with a single line on the command line (or a few lines of python code) and a user-supplied CSV file (see SI for some examples). In the following, we discuss specialty options introduced since its first release.

**Table 1:** Selected Published Studies Based on Chemprop

ref	Year	Prediction	ref	Year	Prediction
(10)	2020	Growth inhibitory activity against *E. coli*; led to an identification of a potential new drug	(51)	2022	Absorption, distribution, metabolism, excretion (ADME) properties for drug discovery
(52)	2021	Chemical synergy against SARS-CoV-2; identified two drug combinations with antiviral synergy in vitro	(53)	2023	Growth inhibitory activity against *A. baumannii*; led to an identification of a potential new drug
(28)	2021	IR spectra of molecules	(54)	2023	Fuel properties
(55)	2021	Atomic charges, Fukui indices, NMR constants, bond lengths, and bond orders	(56)	2023	Critical properties, acentric factor, and phase change properties
(57)	2021	Lipophilicity	(58)	2023	Molecular optical peaks
(59)	2022	Reaction rates and barrier heights	(60)	2023	Lipophilicity
(61)	2022	Barrier heights of reactions	(62)	2023	Solvent effects on reaction rate constants
(29)	2022	Molecular optical peaks	(63)	2023	Vapor pressure in the low volatility regime
(64)	2022	Solvation free energy, solvation enthalpy, and Abraham solute descriptors	(65)	2023	Molecular optical peaks and partition coefficients for closed-loop active learning
(66)	2022	Solid solubility of organic solutes in water and organic solvents	(67)	2023	Senolytic activity of compounds to selectively target senescent cells
(68)	2022	Activity coefficients	(69)	2023	Toxicity measurements using 12 nuclear receptor signaling and stress response pathways

### 3.1 Additional Features

Chemprop can take additional features at the molecule-, atom-, or bond-level as input. While Chemprop often generates accurate models without requiring any input beyond the SMILES, it has been shown that outside information added as additional features can further improve performance.^36,64^ Users can provide their custom additional features by adding keywords and paths to the data files containing the features. See SI for command-line arguments and details.

### 3.2 Multimolecule Models

Chemprop can also train on a data set containing more than one molecule as input. For example, when properties related to solvation need to be predicted, both a solute and a solvent are required as input to the model. Users can provide multiple molecules as inputs to Chemprop. When multiple molecules are used, by default Chemprop trains a separate D-MPNN for each molecule (Figure S1a). If the option --mpn_shared is specified, then the same D-MPNN is used for all molecules (Figure S1b). The embeddings of the different molecules are then concatenated prior to the FFN. Note that the current implementation of multiple molecules in Chemprop does not ensure permutational invariance toward the input molecules. This is suited to situations where the input molecules have different roles, e.g., molecule 1 = solute, molecule 2 = solvent. For additional input information and a figure depicting the multimolecule model structure, see SI.

### 3.3 Reaction Support

**Figure 2:**
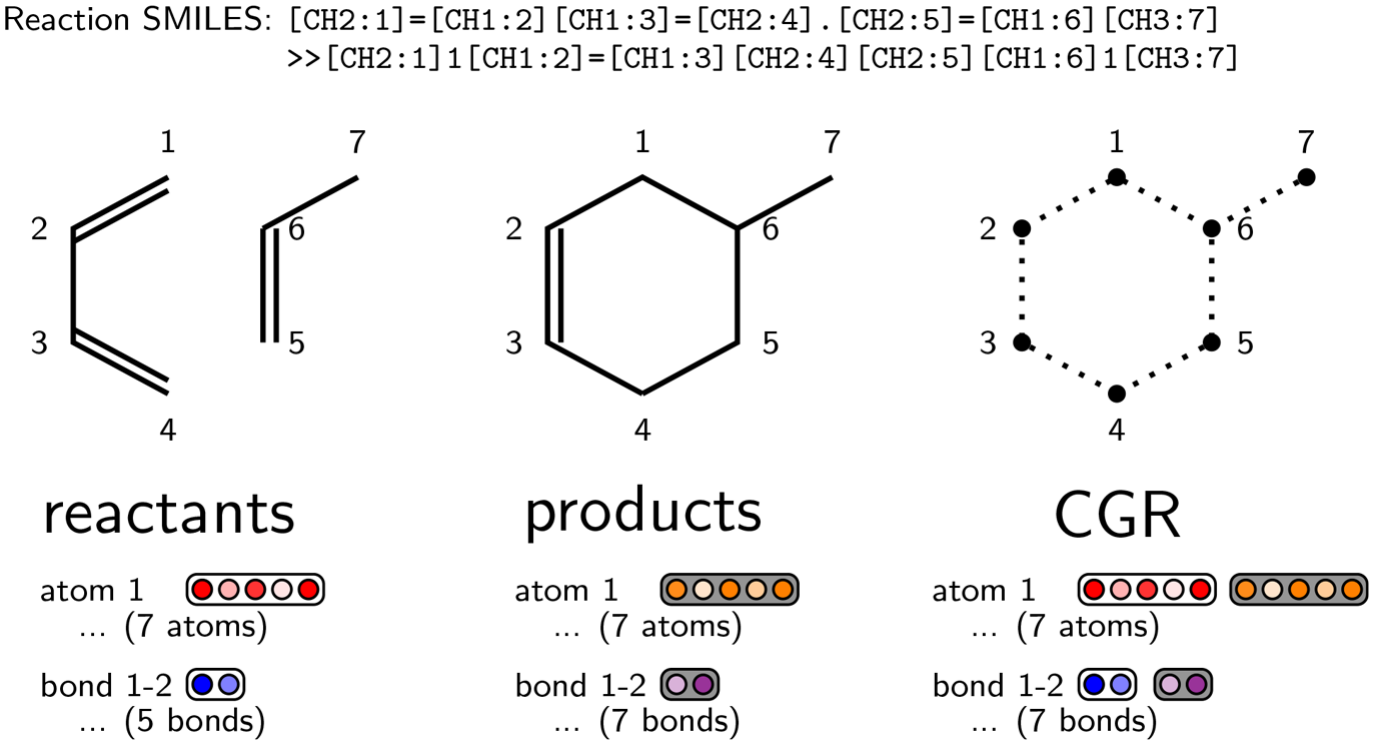
Construction of the condensed graph of reaction (CGR) of an example reaction. The vertices and edges are obtained as the union of the respective reactant and product vertices and edges. The features are obtained as a combination of the reactant (white background) and product (gray background) features for atoms and bonds.

Chemprop supports the input of atom-mapped reactions, i.e., pairs of reactants and products SMILES connected via the “≫” symbol,by using the keyword --reaction. The pair of reactants and products is transformed into a single pseudomolecule, namely, the condensed graph of reaction (CGR), and then passed to a regular D-MPNN block. The construction of a CGR within Chemprop is described in detail in ref (59) and summarized in the following. In general, the input of a reaction vs a molecule only affects the setup of the graph object and its initial features, but not any other part of the architecture. The graph of a reaction has a different set of edges *E* as the graph of a molecule, as shown in Figure 2 for an example Diels–Alder reaction. To build the CGR pseudomolecule, the set of atoms is obtained as the union of the sets of atoms in the reactants and products. Similarly, the set of bonds is obtained as the union of the sets of bonds in the reactants and products. Once constructed, the CGR is passed through the D-MPNN and other model architecture components in the same way a molecular graph would. Optionally, Chemprop accepts an additional molecule object as input, such as a solvent, a reagent, etc. which is passed to its own D-MPNN similar to the multimolecule model. The output of the reaction D-MPNN and molecule D-MPNN is concatenated after atomic aggregation, before the FFN. This option is available via the reaction_solvent keyword. See SI for further commandline options and details.

### 3.4 Spectra Data Support

Chemprop supports the prediction of whole-spectrum properties for molecules. An initial version of this capability was discussed in ref (28) for use with IR absorbance spectra. Targets for the spectra data set type are composed of an array of intensity values, set at fixed bin locations typically specified in terms of wavelengths, frequencies, or wavenumbers. Spectral Information Divergence was originally developed as a method of comparing spectra to reference databases^70^ and is adapted in Chemprop to be used as a loss function, considering the deviation of the spectrum as a whole rather than independently at each bin location. The treatment of spectra can handle targets with gaps or missing values within a data set. With the expectation that spectra will often be collected in systems where a portion of the range will be obscured or invalid (e.g., from solvent absorbance), Chemprop can create exclusion regions in specified spectra where no predictions are provided and targets are ignored for training purposes.

### 3.5 Latent Representations

Graph neural networks enable learning both molecular representation and property end-to-end directly from the molecular graph. As detailed above in Section 2, the learned node representations are aggregated into a molecule-level representation after the message-passing phase, which we refer to as the “learned fingerprint.” This embedding is then further fed into the FFN network. Within the FFN, we consider the final hidden representation, which we refer to as the “ffn embedding”. Both of these vectors are latent representations of a molecule as it relates to a particular trained model. Molecule latent representations can be useful for data clustering or used as additional features in other models. Chemprop supports the calculation of either from a trained model for a given set of molecules.

### 3.6 Loss Function Options

Chemprop can train models according to many common loss functions. The loss functions available for a given task are determined by the data set type (regression, classification, multiclass, or spectra). Regression models can be trained with mean squared error (MSE), bounded MSE (which allows inequalities as targets), or negative log-likelihood (NLL) based on prediction uncertainty distributions consistent with mean-variance estimation (MVE)^71^ or evidential uncertainty.^72^ Classification tasks default to the binary cross entropy loss and have additional options of Matthews correlation coefficient (MCC) and Dirichlet (evidential classification).^73^ Cross entropy and MCC are also available for multiclass problems. There are two options available for training on spectra: spectral information divergence (SID)^70^ and first-order Wasserstein distance (a.k.a. earthmover’s distance).^74^ Loss functions must be differentiable since they are used to calculate gradients that update the model parameters, but Chemprop also provides the option to use several nondifferentiable metrics for model evaluation.

### 3.7 Transfer Learning

Transfer learning is a general strategy of using information gained through the training of one model to inform and improve the training of a related model. Often, this strategy is used to transfer information from a previously trained model of a large data set to a model of a small data set in order to improve the performance of the model of the small data set. The simplest method of transfer learning would be taking predictions or latent representations from one model and supplying them as additional features to another model (Sections 3.1 and 3.5).

**Figure 3:**
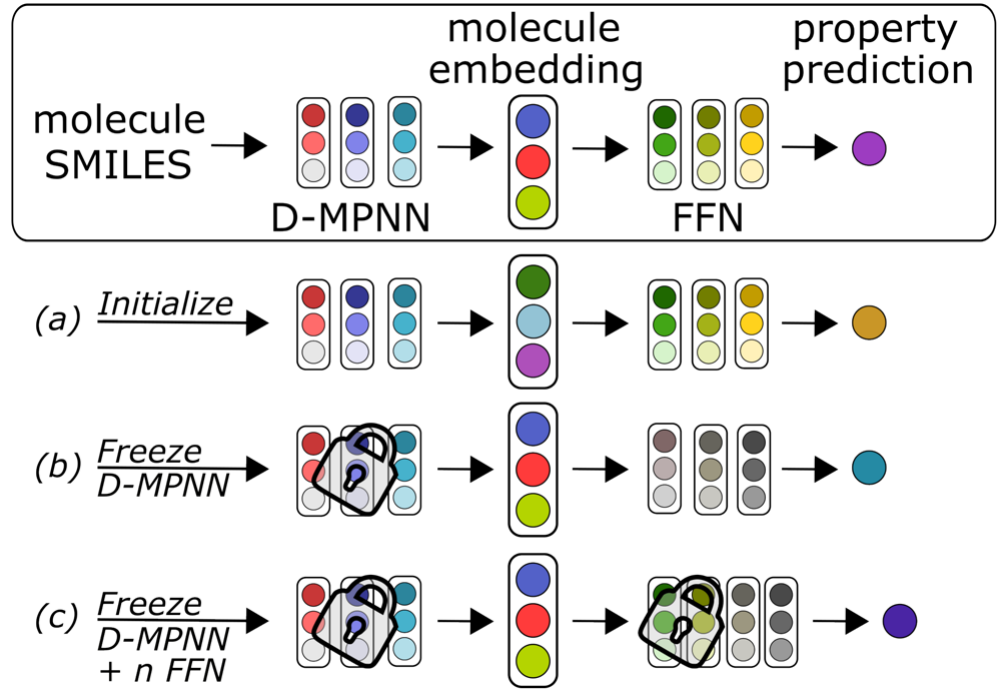
Options to transfer model parameters from a pretrained model (squared) to a new model, by (a) initializing the new model parameters or by freezing the (b) D-MPNN layers and (c) *n* FFN layers.

In Chemprop, different strategies are available to transfer learned model parameters from a previously trained model to a new model as a form of transfer learning, as shown in Figure 3. A pretrained model may be used to initialize a new model with normal updating of the transferred weights in training. Alternatively, parameters from the transferred model can be frozen, holding them constant during training. Freezing parameters always include the D-MPNN weights but can be specified to include some FFN layers as well. See SI for the corresponding arguments.

### 3.8 Hyperparameter Optimization

Chemprop provides a command-line utility, allowing for the simple initiation of hyperparameter optimization jobs. Options for the hyperparameter job such as how many trials to carry out and which hyperparameters to include in the search (options in Table S3) can be specified with simple command-line arguments. The optimization initially uses randomly sampled trials, followed by targeted sampling using the Tree-structured Parzen Estimator algorithm.^75,76^

Hyperparameter optimization is often the most resource-intensive step in model training. In order to search a large parameter space adequately, a large number of trials is needed. Chemprop allows for parallel operation of multiple hyperparameter optimization instances, removing the need to carry out all trials in series and reducing the wall time needed to perform the optimization significantly. See SI for the available hyperparameter options and other details.

### 3.9 Uncertainty Tools

Chemprop includes a variety of popular uncertainty estimation, calibration, and evaluation tools. The estimation methods include deep ensembles,^77^ dropout,^78^ mean-variance estimation (MVE),^71^ and evidential,^72^ as well as a special version of ensemble variance for spectral predictions,^28^ and the inherently probabilistic outputs of classification models. After estimating the uncertainty in a model’s predictions, it is often helpful to calibrate these uncertainties to improve their performance on new predictions. We provide four such methods for regression tasks (*z*-scaling,^79^ t-scaling, Zelikman’s CRUDE,^80^ and MVE weighting^81^) and two for classification (single-parameter Platt scaling^82^ and isotonic regression^83^). In addition to standard metrics such as RMSE, MAE, etc. for evaluating predictions, Chemprop also includes several metrics specifically for evaluating the quality of uncertainty estimates. These include negative log likelihood, Spearman rank correlation, expected normalized calibration error (ENCE),^84^ and miscalibration area.^84^ Any valid classification or multiclass metric used to assess predictions can also be used to assess uncertainties.

### 3.10 Atom/Bond-Level Targets

Chemprop supports a multitask constrained D-MPNN architecture for predicting atom- and bond-level properties, such as charge density or bond length. This model enables a D-MPNN to be trained on multiple atomic and bond properties simultaneously, though unlike molecular property targets, they do not share a single FFN. Optionally, an attention-based constraining method may be used to enforce that predicted atomic or bond properties sum to a specified molecular net value, such as the overall charge of a molecule. An initial, more limited version of this capability was developed in ref (55). For details on the input formats to be used for atom/bond targets, both for training and for inference, see SI.

## 4 Benchmarking

See SI for general performance benchmarks, benchmarks using specific Chemprop features (atom/bond-level targets, reaction support, multimolecule models, spectra prediction, and uncertainty estimation), and system timing benchmarks.

## 5 Conclusion

We have presented the software package Chemprop, a powerful toolbox for machine learning of the chemical properties of molecules and reactions. Significant improvements have been made to the software since its initial release and study,^36^ including the support of multimolecule properties, reactions, atom/bond-level properties, and spectra. Additionally, several state-of-the-art approaches to estimate the uncertainty of predictions have been incorporated as well as pretraining and transfer learning procedures. Furthermore, the code now offers a variety of customization options, such as custom atom and bond features, a large variety of loss functions, and the ability to save the learned feature embeddings for subsequent use with different algorithms. We have showcased and benchmarked Chemprop on a variety of example tasks and data sets and have found competitive performances for molecular property prediction compared to other approaches available on public leaderboards. In summary, Chemprop is a powerful, fast, and convenient tool to learn conformation-independent properties of molecules, sets of molecules, or reactions.

## Data Availability

Chemprop, including all features described in this paper, is available under the open-source MIT License on GitHub, github.com/chemprop/chemprop. An extensive documentation including tutorials is available online,^85^ including a workshop on YouTube.^86^ Scripts and data splits to fully reproduce this study are available on GitHub, github.com/chemprop/chemprop_benchmark, and on Zenodo, doi.org/10.5281/zenodo.8174267, respectively.

## Supporting Information Available

Additional software details and usage examples, data set and data handling details for benchmarking, and results of software benchmarks (general performance, feature demonstrations, timing) (PDF)
