# Supplementary material for: Chemprop: A Machine Learning Package for Chemical Property Prediction
Source: J Chem Inf Model. 2023 Dec 26;64(1):9–17. doi: 10.1021/acs.jcim.3c01250 (PMC10777403; doi:10.1021/acs.jcim.3c01250)
Supplement: Supplementary file 1 — ci3c01250_si_001.pdf [file ci3c01250_si_001.pdf]

Supporting Information

Chemprop: A Machine Learning Package for Chemical Property Prediction

Esther Heid,[†,‡] Kevin P. Greenman,[†] Yunsie Chung,[†] Shih-Cheng Li,[†,¶] David E. Graff,[†,§] Florence H. Vermeire,[†,‖] Haoyang Wu,[†] William H. Green,[†] and Charles J. McGill[*,†,⊥]

†*Department of Chemical Engineering, Massachusetts Institute of Technology, Cambridge, Massachusetts 02139, United States*

‡*Institute of Materials Chemistry, TU Wien, 1060 Vienna, Austria*

¶*Department of Chemical Engineering, National Taiwan University, Taipei 10617, Taiwan*

§*Department of Chemistry and Chemical Biology, Harvard University, Cambridge, Massachusetts 02138, United States*

‖*Department of Chemical Engineering, KU Leuven, Celestijnenlaan 200F, B-3001 Leuven, Belgium*

⊥*Department of Chemical and Life Science Engineering, Virginia Commonwealth University, Richmond, Virginia 23284, United States*

E-mail: mcgillc2@vcu.edu

Contents

1 Additional Model Details **4**

1.1 Example commands 4

1.2 Additional features 4

1.3 GPU support 8

1.4 Regularization 8

1.5 Multi-molecule models 9

1.6 Reaction support 10

1.7 Hyperparameter optimization 11

1.8 Atom/bond-level targets 13

2 Benchmark methods **14**

2.1 Hyperparameter tuning 14

2.2 Datasets 15

2.2.1 MoleculeNet & OGB 15

2.2.2 SAMPL 16

2.2.3 Atom/bond-level targets 17

2.2.4 Reaction barrier heights 19

2.2.5 UV/Vis absorption 20

2.2.6 IR spectra 20

2.2.7 HOMO-LUMO gaps 20

3 Benchmark results **21**

3.1 Model Performance: General benchmarking 21

3.1.1 MoleculeNet & OGB 21

3.1.2 PCQM4Mv2 23

3.1.3 SAMPL 23

3.2 Model Performance: Specific feature demonstrations 24
3.2.1 Atom/bond-level targets 24
3.2.2 Reaction barrier heights 26
3.2.3 UV/Vis absorption 27
3.2.4 IR spectra 28
3.2.5 Uncertainty estimation for QM9 gap 29
3.3 Timing 29

1 Additional Model Details

1.1 Example commands

To train a default model on the ESOL solubility dataset [1] which is distributed with Chemprop as CSV file, and save the results to the folder "checkpoint", run

```
chemprop_train --data_path data/delaney.csv
--dataset_type regression --save_dir checkpoint
--save_smiles_splits
```

on the command line after installation of Chemprop following the instructions on Github. [2] This splits the data randomly into training, validation and test sets in the ratio 80/10/10, trains a default model and computes the performance on the test set. To compute predictions using an already trained model, run

```
chemprop_predict --checkpoint_dir checkpoint
--test_path checkpoint/fold_0/test_smiles.csv
--preds_path checkpoint/test_preds.csv
```

which takes the previously generated test set, computes predictions using all models in the checkpoint folder and saves them to the indicated path. For the use of Chemprop within a Python script or a graphical web interface, as well as many options to customize the model, data splits, and performance metrics please consult the instructions on Github [2] or the Chemprop documentation. [3] Hyperparameter optimization can be performed with similar commands, as detailed in the Discussion of Features section.

1.2 Additional features

Users can provide their custom additional features by adding keywords and paths to the data files containing the features.

For molecule-level features x_m, a path to the features can be specified using the keyword `--features_path PATH/TO/FEATURES`. The provided molecular features are concatenated to the learned molecular embedding prior to the FFN network. The features can be provided as a numpy `.npy` file or CSV file. For both file formats, the features must be in the same order as the SMILES strings in the data file. The features file should not contain the SMILES strings, since features will be associated with the corresponding molecule based on the ordering in the file. The features file should contain numerical values, with columns corresponding to different features and rows corresponding to molecule data points. By default, provided features are normalized unless the flag `--no_features_scaling` is used.

For additional atomic features x_v, the path to the features can be provided using the keyword `--atom_descriptors_path PATH/TO/FEATURES`. The supported file formats include `.npz`, `.pkl`, and `.sdf`. Two options are available to select in which way atom descriptors are used. The option `--atom_descriptors descriptor` concatenates the additional features to the embedded atomic features after the D-MPNN. On the other hand, the option `--atom_descriptors feature` concatenates the features to the initial atomic feature vectors prior to the D-MPNN, such that they can be used during message-passing. Additional bond-level features can be provided via `--bond_descriptors_path PATH/TO/FEATURES` in the same format as the atom-level features. Similarly, users must choose in which way bond descriptors are used. The option `--bond_descriptors descriptor` concatenates the new bond-level features to the embedded bond features after the D-MPNN, which can only be used for bond-level property prediction, while the option `--bond_descriptors feature` concatenates the new features with the default bond feature vectors before the D-MPNN.

Users must ensure that the order of additional atom and bond features match the atom and bond ordering in the RDKit molecule object. If users wish to only use their custom features instead of the default features, the keywords `--overwrite_default_atom_features` and `--overwrite_default_bond_features` can be used to overwrite the default atom and bond features, respectively. The overwrite option is only available when the additional fea-

tures are used as `feature`. Similar to the molecular-level features, the atom- and bond-level features will be normalized automatically by default. This can be disabled with the options `--no_atom_descriptor_scaling` and `--no_bond_descriptor_scaling`.

The inputs of atom and bond features can be provided via three file formats:

- `.npz` format

  Atomic descriptors are saved as 2D array ([number of atoms x number of descriptors]) for each molecule in the exact same order as the SMILES strings in the data file. Similarly, bond descriptors are saved as 2D array ([number of bonds x number of descriptors]). For example:

  ```
  np.savez('descriptors.npz', *descriptors)
  ```

  where `descriptors` is a list of atomic or bond descriptors in 2D array in the order of molecules in the training/predicting datafile.

- `.pkl`/`.pckl`/`.pickle` format

  It contains a pandas dataframe with SMILES as index and a numpy array of descriptors as columns. For example:

Table S1: Custom atomic features for each atom provided in 1D arrays.

**smiles**	**descriptors**
CCOc1ccc2nc(S(N)(=O)=O)sc2c1	[0.6377, 0.7075...]
CCN1C(=O)NC(c2ccccc2)C1=O	[0.0958, 0.6521...]

Table S2: Multiple atomic features for each atom provided in multiple 1D arrays.

**smiles**	**desc1**	**desc2**
CCOc1ccc2nc(S(N)(=O)=O)sc2c1	[0.6377, 0.7075...]	[0.8266, 0.8964...]
CCN1C(=O)NC(c2ccccc2)C1=O	[0.0958, 0.6521...]	[0.2847, 0.8410...]

- `.sdf` format

Each molecule is presented as a mol block in the file. Descriptors should be saved as entries for each mol block in the format of comma separated values. Each molecule must has an entry named SMILES that stores the SMILES string. For example:

```
CHEMBL1308_loner5
     RDKit          3D


  6  6  0  0  1  0  0  0  0  0999 V2000
   -0.7579   -0.5337   -2.8744 C   0  0  0  0  0  0  0  0  0  0
       0  0
   -0.2229   -1.3763   -1.7558 C   0  0  0  0  0  0  0  0  0  0
       0  0
   -0.0046   -1.0089   -0.4029 C   0  0  0  0  0  0  0  0  0  0
       0  0
    0.4824   -2.0104    0.3280 N   0  0  0  0  0  0  0  0  0  0
        0  0
    0.5806   -3.0317   -0.5484 N   0  0  0  0  0  0  0  0  0  0
        0  0
    0.1735   -2.6999   -1.8031 C   0  0  0  0  0  0  0  0  0  0
        0  0
  1  2  1  0
  2  6  2  0
  2  3  1  0
  3  4  2  0
  4  5  1  0
  5  6  1  0
M  END
>  <desc1>  (1)
-8.568031e-05,0.0001865207,-0.0002012379,-5.054658e
   -05,0.0002148434,-0.0003503839,1.970448e-05,3.081137e
```

```
-05,2.997883e-05,9.446278e-05,-7.194711e-05,0.0001527364

> <desc2> (1)
5.462954e-05,-2.415399e-06,0.0001044788,-2.274438e
-05,0.0001698836,5.206409e-06,4.5825e-06,-8.882181e
-07,-1.08787e-05,2.993307e-05,-4.069051e-06,1.338413e-05

> <SMILES> (1)
Cc1cnnHc1

$$$$
```

1.3 GPU support

The PyTorch backend of Chemprop enables seamless GPU acceleration of both model training and inference. The acceleration of training and inference processes when used with a GPU can be significant, as shown later in Section 3.3. This feature is enabled by default on machines possessing a CUDA-enabled GPU, but it may be turned off via the `--no-cuda` argument on the command line. For machines with multiple GPUs, Chemprop will use the default GPU as PyTorch (typically the GPU at index 0). It is possible to specify the kth GPU by either passing `--gpu <k>` on the command or setting the `CUDA_VISIBLE_DEVICES` environment to show only device `k` (i.e., executing `CUDA_VISIBLE_DEVICES=k` prior to running Chemprop).

1.4 Regularization

Chemprop has two builtin forms of regularization, intended to help reduce overfitting in trained models. These two regularization techniques were present in the initial release of Chemprop and remain an important contributor to model quality. The first form of regu-

larization is called early stopping. With early stopping, the performance of the model on the validation set is calculated at the end of each epoch. The version of the model that is stored at the end of training is the one saved at the end of the best scoring epoch. This has the effect of discarding later epochs of training where the model would be overfitting to the training data, continuing to improve the training loss at the cost of hurting performance on the validation and test sets. Contrary to what the name implies, early stopping as implemented in Chemprop does not shorten the amount of time needed for training.

The second form of regularization is called dropout. During training, dropout regularization will randomly zero out a fraction of the latent variables for that forward pass. This practice has been shown to reduce overfitting and lead to higher quality latent variables.[4] The level of dropout regularization can be specified using the option `--dropout <p>` where p is the dropout probability. By default, dropout is inactive. We have observed dropout to be a helpful addition to models in a variety of contexts and recommend that users include it in their choices of hyperparameters.

1.5 Multi-molecule models

The number of molecules N is specified with the keyword `--number_of_molecules`. For the example of a solute-solvent pair $N = 2$. To train a new model using multiple molecules as an input, the SMILES string of each molecule must be provided as a separate column in the input CSV file. If N molecules are used, Chemprop assumes that the SMILES strings are located in the first N columns by default. Alternatively, the names of the specific columns containing the SMILES of the different molecules can be specified using the `--smiles_columns <column_1> ...` option. The embedding of multiple molecules in Chemprop can be done in two different ways as schematically represented in Figure S1 for $N = 2$. When multiple molecules are used, by default Chemprop trains a separate D-MPNN for each molecule (Figure S1a). If the option `--mpn_shared` is specified, the same D-MPNN is used for all molecules (Figure S1b). In both cases, the D-MPNN of each molecule uses the

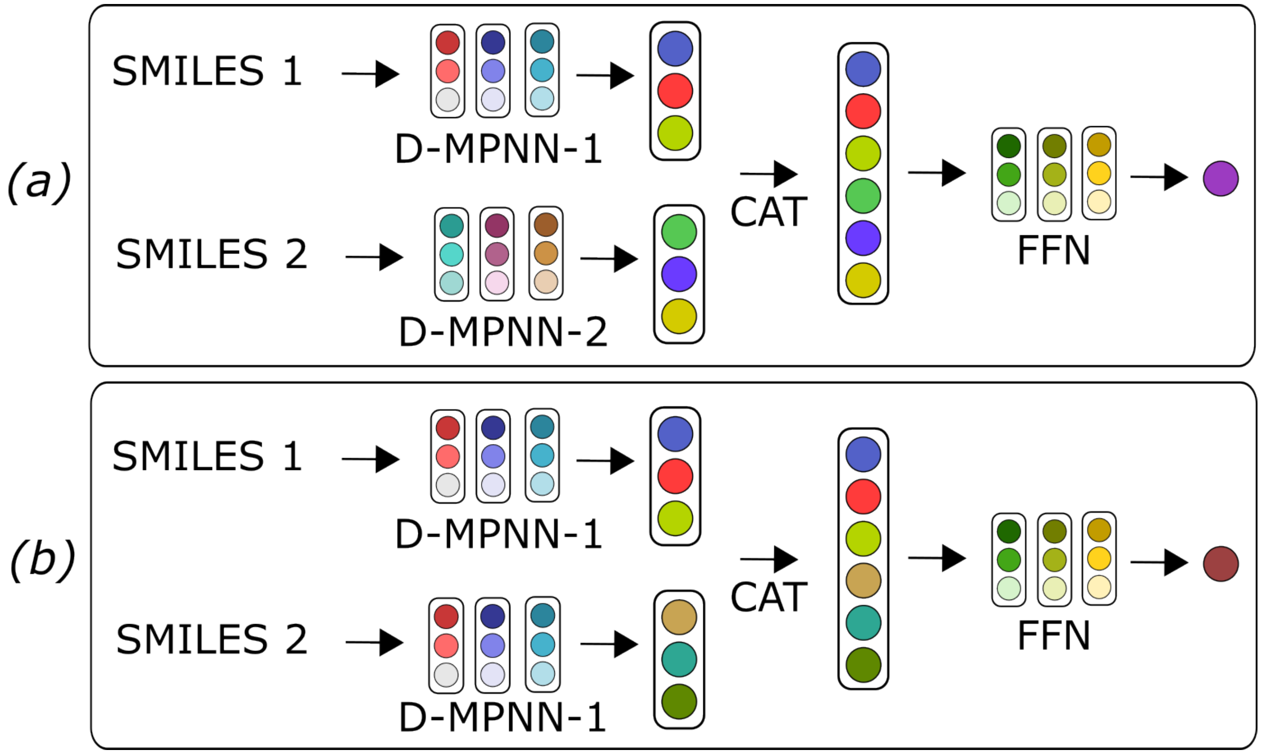

Figure S1: Example of how two molecules ($N = 2$) can be embedded in Chemprop. a) A separate D-MPNN is used for each molecule or b) the same D-MPNN is used. After embedding, the different molecular vectors are concatenated (CAT) and used as input to the feed forward network (FFN) for property prediction.

same hyperparameters. The embeddings of the different molecules are then concatenated prior to the FFN. Note that the current implementation of multiple molecules in Chemprop does not ensure permutational invariance towards the input molecules. This is suited to situations where the input molecules have different roles, e.g. molecule 1 = solute, molecule 2 = solvent. For additional input information and a figure depicting the multi-molecule model structure, see the *SI*.

1.6 Reaction support

The initial atom and bond feature vectors in the CGR contain information on both the reactant and product features. Whenever information is not available, e.g. because a bond did not exist in either the reactants or products, the features are set to zero. A simple concatenation of reactant and product features can be used to obtain the pseudomolecule features

(keyword `--reaction_mode reac_prod`). Since the atomic number does not change upon reaction, its one-hot encoding is not repeated in the second part of the feature vector. For many reaction properties the change in the local structure upon reaction, i.e. the difference between reactants and products, is very informative. Since neural networks are known to not perform well for adding and subtraction operations, we also provide options to include the difference in properties directly. Namely, one can concatenate the difference in atom and bond features with the reactant properties (keyword `--reaction_mode reac_diff`, default) or with the product properties (keyword `--reaction_mode prod_diff`). Further, we provide options to automatically balance imbalanced reactions (via setting `--reaction_mode` to `reac_prod_balance`, `reac_diff_balance` or `prod_diff_balance`), which is useful if a dataset contains both balanced and imbalanced reactions. For datasets containing only balanced reactions, or only imbalanced reactions, e.g. where leaving groups are never specified, this is usually not necessary. Optionally, Chemprop accepts an additional molecule object as input, such as a solvent, or a reagent, etc. which is passed to its own D-MPNN similar to the multi-molecule model. The output of the reaction D-MPNN and molecule D-MPNN is concatenated after atomic aggregation, before the FFN. This option is available via the `reaction_solvent` keyword. The size of the molecule D-MPNN can be varied with the keywords `hidden_size_solvent` and `depth_solvent`. We note that this additional solvent or reagent needs to be passed as a separate SMILES column in the input CSV file. Reagents passed within the reaction SMILES string (between the `>` symbols) are disregarded. An example of the reaction-solvent model can be found in Ref. 5, in which a Chemprop model is trained to predict kinetic solvent effects for a diverse range of reactions and solvents.

1.7 Hyperparameter optimization

Hyperparameter optimization is a key step when building ML models that can lead to significant gains in model performance. Given the sheer size of the search space with deep learning models, this step can often be operationally challenging. Chemprop provides a

Table S3: Searchable hyperparameters using chemprop_hyperopt.

Keyword	Description
activation	the activation function used after each linear layer, when necessary
aggregation	the aggregation function used when constructing a molecule-level representation from node-level representations
aggregation_norm	the normalization factor if using norm aggregation
batch_size	the minibatch size
depth	the number of message-passing iterations
dropout	the dropout probability after each layer in both the D-MPNN encoder and FFN
ffn_hidden_size	the size of each hidden layer in the FFN
ffn_num_layers	the number of layers in the FFN
hidden_size	the message size in the D-MPNN encoder
linked_hidden_size	the size of *both* the messages in the D-MPNN encoder and the hidden layers in the FFN. This argument is overridden by either hidden_size or ffn_hidden_size
max_lr	the maximum learning rate used in the learning rate scheduler
init_lr	the initial learning rate expressed as the ratio of init_lr to max_lr
final_lr	the final learning rate expressed as the ratio of final_lr to max_lr
warmup_epochs	the number of epochs over which to ramp up the learning rate up from init_lr to max_lr expressed as a fraction of the total training epochs
basic	search over depth, ffn_num_layers, dropout, and linked_hidden_layers
learning_rate	search over init_lr, max_lr, final_lr, and warmup_epochs
all	all of the above hyperparameters

command line utility, chemprop_hyperopt, that automates this process by removing the need to manually define the search space of hyperparameters. Users can simply supply a list of keywords from which to build a hyperparameter search space (Table S3). The number of trials of hyperparameter combinations to be tested can be set using the --num_iters argument. By default, the search space will first be randomly sampled for num_iters/2 trials before switching to targeted sampling via the tree-structured Parzen estimator algorithm[6,7] for the remaining trials. The number of random trials to be used can be changed by setting --startup_random_iters to a value less than num_iters.

Hyperparameter optimization can be the most resource-intensive step in model training. In order to search a large parameter space adequately, a large number of trials would be needed. Chemprop allows for parallel operation of multiple hyperparameter optimization instances, so that the entire set of trials does not need to be run in series. Parallel operation can be achieved by setting the location of trial checkpoint files with --hyperopt_checkpoint_dir to be a single shared location for multiple hyperparameter optimization instances. This allows for multiple instances of the program to share and contribute to the same trial history, reducing the wall time needed to perform hyperparameter optimization significantly.

1.8 Atom/bond-level targets

The input is provided as a CSV file. The targets of atomic properties must be a 1D list in the same order as the atoms in the RDKit[8] molecule object. The bond properties can either be a 2D list of shape $n \times n$, where n is the number of atoms, or a 1D list in the same order as the bonds in the RDKit molecule object. An example file with both atomic and bond targets is shown in Table S4. It is also important to note that Chemprop can autodetect whether a target should be an atomic or bond target.

Table S4: Example input file for atom- and bond-level property prediction. The value of hirshfeld_charges is presented as a 1D list, while the value of bond_index_matrix is presented as a 2D list.

**smiles**	**hirshfeld_charges**	**bond_index_matrix**
CNC(=S)N/N=C/c1c(O)ccc2ccccc12	[-0.0266, -0.0755...]	[[0.0000, 0.9595...] ...[0.0000, 0.0000...]]
O=C(NCCn1cccc1)c1cccc2ccccc12	[-0.2924, 0.1702...]	[[0.0000, 1.6334...] ...[0.0059, 0.0009...]]
C=C(C)[C@H]1C[C@@H]2OO[C@H]1C=C2C	[-0.1017, 0.0123...]	[[0.0000, 1.9083...] ...[0.0000, 0.0000...]]
OCCCc1cc[nH]n1	[-0.2684, 0.0276...]	[[0.0000, 0.9446...] ...[0.0000, 0.0000...]]
CC(=N)NCc1cccc(CNCc2ccncc2)c1	[-0.0832, 0.1150...]	[[0.0000, 1.0036...] ...[0.0005, 0.0009...]]

Alternatively, the `--keeping_atom_map` option can be used if users wish to use atom-mapped SMILES. To apply the summation constraint to properties for each molecule, a path to the constraints can be specified using the keyword `--constraints_path PATH/TO/CONSTRAINTS` in the same order as the SMILES strings in the data file. Different constraints should be separated into different columns with a header row and one row per molecule, and the file should not contain the SMILES string. Which targets will be constrained is controlled by the names of the tasks in the constraint file header. For properties without constraints, the atomic or bond embeddings will be linked with FFN layers. Conversely, for properties with sum constraints, attention-based layers will also be constructed for each target.[9] By default, the atom tasks share FFN weights, and bond tasks share FFN weights so that the FFN

weights might benefit from multitask training. The argument `--no_shared_atom_bond_ffn` can be used if users want to train the FFN weights for each task independently. The argument `--no_adding_bond_types` will let the bond types of each bond determined by RDKit molecules not be added to the output of bond targets. For attention-based constraining, the argument `--weights_ffn_num_layers` can be used to change the number of layers in the FFN for determining weights used to correct the constrained targets (default 2).

2 Benchmark methods

In the following, we describe the hyperparameter tuning procedure for our benchmark studies as well as the source, splitting routines, and further information on all benchmark datasets employed in this study.

2.1 Hyperparameter tuning

Training of benchmark models was carried out using hyperparameters optimized for each task. Throughout the remainder of this study, we classify datasets as small/large if they contain less/more than 10k data points in total. Models trained on small datasets were optimized for hyperparameters using 100 search iterations, whereas models trained on large datasets were optimized for only 30 iterations. During hyperparameter tuning and final model training, we trained for 200/50 epochs for small/large datasets. All models were trained on a single data split with an ensemble size of 5 during the final training, and without ensembling for hyperparameter tuning. During hyperparameter tuning, we optimized for the number of message passing steps, the hidden size during message passing, the number of layers of the feed forward neural network, as well as its hidden size, and the dropout ratio. For small datasets, furthermore the learning rate (initial, final and maximum), warum-up period and batch size were optimized. For both hyperparameter tuning and model production, scaled sums were used to aggregate atomic into molecular feature vectors. All other

parameters were left at their default values.

2.2 Datasets

The benchmarking datasets used in this study are listed in Table S5. All datasets are publicly available from the literature as described in the following. Various evaluation metrics are used to assess the performance of the Chemprop models on each dataset and against other models previously reported in the literature:

- ROC-AUC: area under the receiver operating characteristic curve
- PRC-AUC: area under the precision-recall curve
- AP: average precision
- MAE: mean absolute error
- RMSE: root-mean-square error
- R^2: coefficient of determination
- SID: spectral information divergence

2.2.1 MoleculeNet & OGB

The HIV and PCBA datasets from MoleculeNet[10] and Open Graph Benchmark (OGB)[11] were selected for classification tasks. Both MoleculeNet and OGB provide a diverse set of benchmark datasets that have been widely used to compare the performance of various machine learning models. They also host public leaderboards that allow us to directly compare our results to other public models. The HIV dataset contains results from an assay designed to detect HIV inhibition for 41,127 compounds. It has been observed that many of the species in the HIV dataset are at risk for assay result artifacts[12], so in the narrowest sense dataset performance should be viewed as a test for the assay result rather

than strictly as a predictor for HIV inhibition. The PCBA dataset includes the 128 biological activities selected from PubChem BioAssay[13] for 437,929 compounds. The datasets were evaluated using the random and scaffold splits that were provided by MoleculeNet and OGB. We adopted the training, validation, and test sets of the scaffold-split HIV data and the random-split PCBA data from MoleculeNet. The scaffold-split PCBA data were adopted from OGB as MoleculeNet did not evaluate the PCBA model on the scaffold split. In all splits, the datasets were split into 80% training, 10% validation, and 10% test sets. For the random-split PCBA, MoleculeNet sets all missing targets to zero (in contrast to OGB), so we report performances for either case, i.e. filled-in zeros for comparability to the MoleculeNet leaderboard, and without filled-in values, to showcase how the observed performance drops when adopting a scaffold split versus a random split.

QM9 is a dataset of DFT calculation values commonly used for chemical model benchmarking. The calculations for this dataset were originally carried out by Ramakrishnan et al.[14] and later distributed as part of the MoleculeNet benchmarks.[10] The dataset is made up of 133,885 molecules with properties and structures calculated at the B3LYP/6-31G(2df,p) level of theory. The molecules were chosen as the set of possible molecules containing up to nine heavy atoms of the types C, N, O, and F. Data sources for QM9 provide 3D coordinates for the atoms in the optimized structures, but we only use molecule SMILES as inputs for model training in this work. QM9 provides 12 target values for each molecule, provided in Table S6. In the MoleculeNet presentation of the properties, atomized versions of the thermochemical properties U0, U298, H298, and G298 are provided alongside the original versions of the properties. In this work, we will use the atomized thermochemical properties. This dataset was randomly divided into 80% training, 10% validation, and 10% test data.

2.2.2 SAMPL

Experimental logP data of the SAMPL6, SAMPL7 and SAMPL9 challenges was downloaded from the SAMPL GitHub repository.[15] SAMPL runs a series of blind challenges for compu-

Table S5: Summary of the benchmarking datasets.

Dataset/Category	Property/Data type	Type	N tasks	N data	Metric(s)	Ref.[a]
MoleculeNet & OGB	HIV (HIV replication inhibition)	Class.	1	41,127	ROC-AUC	10
	PCBA (biological activities)	Class.	128	437,929	PRC-AUC, AP	10,11
	QM9 (DFT calculated properties)	Regr.	12	133,885	MAE, RMSE	10,14
SAMPL	logP	Regr.	1	23,469[b]	RMSE	15,16
Atom/bond-level targets	Quantum mechanical descriptors	Regr.	6	136,219	MAE, RMSE	9
	Bond dissociation enthalpy	Regr.	1	42,577	MAE	17,18
	Partial charge	Regr.	1	130,267	MAE, RMSE	19
Reaction barrier heights	E2	Regr.	1	1264	MAE	20–23
	S_N2	Regr.	1	2361	MAE	20–23
	Cycloaddition	Regr.	1	5269	MAE	24
	RDB7	Regr.	1	23,852[c]	MAE	25
	RGD1-CNHO	Regr.	1	353,984[c]	MAE	26
UV/Vis Absorption	UV/Vis peak absorption wavelength	Regr.	1	26,395	MAE, RMSE, R^2	27–30
IR Spectra	Spectra between 400 and 4000 cm^{-1}	Spectra	1	8,754	SID	31
PCQM4MV2	HOMO-LUMO gap	Regr.	1	3,452,151	MAE, RMSE	32

[a] References for the data and data splits.
[b] The size of the training set. The SAMPL6, SAMPL7, and SAMPL9 data are used as a test set.
[c] Including reverse reactions.

tational chemistry, providing the identity of test molecules for which predictions of physicochemical properties, among them the water-octanol partition coefficients, can be submitted using quantum-mechanics, molecular mechanics, or empirical models. In this work, we build an empirical model based on Chemprop, which we train on a publicly available dataset of logP measurements from Ref. 16. Molecules present in the SAMPL challenges were removed from the logP training dataset. The remaining 23,469 data points were randomly split into 80% training, 10% validation, and 10% test data. The test data was used to obtain a measure of performance for the literature dataset. We then retrained a production model on the full dataset (no validation or test data) using the best hyperparameters and number of epochs identified earlier, with which we made predictions for the three SAMPL challenges.

2.2.3 Atom/bond-level targets

To predict atom-level and bond-level targets, we selected three benchmark datasets. The framework we used to predict atomic and bond properties in Chemprop was based on modifications made to the approach developed by Guan et al.[9] They published a dataset of

Table S6: Target values present in the QM9 dataset, presented with the target labels used in the dataset.

Label	Units	Description
mu	D	Dipole moment
alpha	α_0^3	Polarizability
HOMO	Ha	Highest occupied molecular orbital energy
LUMO	Ha	Lowest unoccupied molecular orbital energy
gap	Ha	Homo-Lumo gap
r2	α_0^2	Electronic spacial extent
ZPVE	Ha	Zero point vibrational energy
U0	kcal/mol	Internal energy at 0 K
U298	kcal/mol	Internal energy at 298.15 K
H298	kcal/mol	Enthalpy at 298.15 K
G298	kcal/mol	Free energy at 298.15 K
Cv	cal/(mol K)	Heat capacity at 298.15 K

quantum mechanical (QM) descriptors for 136,219 organic molecules with atom types H, C, O, N, F, S, Cl, Br, B, I, P, and Si. This dataset included atomic charges, Fukui indices, NMR shielding constants, bond length, and bond orders. Those molecules were optimized using GFN2-xTB and subjected to population analysis using the B3LYP/def2-SVP level of theory. We used this dataset to evaluate the performance of different implementations, randomly splitting it into 80% training, 10% validation, and 10% test data.

For benchmarking, we also used the BDE-db dataset from St. John et al.[17] This dataset contains bond dissociation enthalpies (BDEs) for 42,577 closed-shell organic molecules with up to 9 heavy atoms of types C, H, O, and N, resulting in 290,664 BDEs. BDEs were calculated using the M06-2X/def2-TZVP level of theory. We used the same data splits as their study,[18] with 40,577 data points as training set and 1000 molecules each in the validation and test sets.

Lastly, we included a dataset of DDEC partial charges, which includes partial charges calculated with different dielectric constants ($\epsilon = 4$ for charges in protein and $\epsilon = 78$ for charges in water).[19] The dataset comprises 130,267 moderate size organic molecules with elements of types C, H, N, O, S, P, F, Cl, Br, and I, curated from ZINC and ChEMBL databases. A small fraction of data in the $\epsilon = 78$ dataset was dropped due to issues with SMILES conversion. We then randomly split the datasets into 80% training, 10% validation, and 10% test data. Two external test sets of 146 organic liquids and 1081 FDA-approved

drugs were used to test the transferability of the models.

2.2.4 Reaction barrier heights

To benchmark Chemprop's reaction functionality, four datasets of computational barrier heights were selected to cover a broad range of dataset size, diversity and quality. Since some of the original publications only report model mean absolute errors, we also report mean absolute errors, although we train on mean squared errors similar to all other benchmarks in this study.

First, E2 and S_N2 reactions originally published in Ref. 20 were used with reaction SMILES as provided in Ref. 21. We used the same split sizes as Ref. 22 and Ref. 23, which are 1440 training and 360 validation data points for S_N2, as well as 800 training and 200 validation data points for E2 with random splits. All other data points were used for testing. We then compared the performance of Chemprop to those reported in Ref. 22 and Ref. 23.

Second, cycloaddition reactions from Ref. 24 were used, with random splits into 80% training, 10% validation and 10% test data. We then compared Chemprop against all models reported in Ref. 33.

Third, the RDB7 dataset, which contains 11,926 high-accuracy reaction barrier heights and enthaplies calculated at CCSD(T)-F12/cc-pVDZ-F12 as provided in Ref. 25. In contrast to the E2, S_N2, and cycloaddition datasets that focus on one specific reaction class, this dataset spans a large range of barrier heights and is used to assess Chemprop's performance on substantially more reaction diversity. We randomly split the data into 80% training, 10% validation and 10% test data and then added reverse reactions to each set.

Fourth, the RGD1-CNHO dataset[26] was used, which comprises the largest and most diverse dataset out of the four, and also the most difficult to learn. We again randomly split the data into 80% training, 10% validation and 10% test data and then added reverse reactions to each set.

2.2.5 UV/Vis absorption

Multi-molecule models are demonstrated using prediction of UV/Vis peak absorption wavelength, a prediction model that involves both the absorbing molecule and the solvent. Our dataset of the peak wavelength of maximum absorption ($\lambda_{max,abs}$) is a combination of several databases[27–30] that were extracted from the experimental literature. There are 26,395 samples across a variety of dye molecule families and solvents. Each sample consists of a dye molecule SMILES, solvent molecule SMILES, and a peak wavelength value. There are no multi-component species of either dyes or solvents. The train-validation-test splits are in 80/10/10 proportions and are constrained to avoid data leakage of highly-correlated measurements of the same dye in multiple solvents.

2.2.6 IR spectra

The dataset used for whole-spectra predictions was collected from infrared absorption spectra made public by NIST.[31] This dataset comprises 8,754 gas-phase spectra, with absorbance magnitudes indicated at 2 cm^{-1} intervals between 400 and 4000 cm^{-1}. The spectra for different molecules have different ranges of collected absorbance and may have regions of missing or excluded values. We randomly split this dataset into 80% training, 10% validation, and 10% test data.

2.2.7 HOMO-LUMO gaps

The PCQM4MV2 dataset is a collection of DFT-calculated molecular HOMO-LUMO gaps, originally collected as part of the PubChemQC project[32] and now curated as part of the Open Graph Benchmark.[11] This dataset contains HOMO-LUMO gaps measured in units of eV for 3,452,151 molecules. A further 294,470 molecules have targets privately held by the Open Graph Benchmark for blinded testing purposes and are not included in the benchmarks performed in this work. For benchmark training, the data we had available was randomly divided into 80% training, 10% validation, and 10% test data. The Open Graph Benchmark

Table S7: Test set metrics for the different targets of QM9. The top grouping of tasks was trained together in a single multitask model. The bottom grouping of results for U0 and gap shows the results for single-task models. The atomized basis of the thermochemical properties U0, U298, H298, and G298 were used for training.

Model	Target	MAE	RMSE
multitask	mu	0.326	0.581
	alpha	0.231	0.523
	HOMO	0.002 40	0.004 17
	LUMO	0.002 38	0.004 07
	gap	0.003 28	0.005 80
	r2	17.4	33.4
	ZPVE	0.000 262	0.000 366
	Cv	0.111	0.222
	U0	1.90	3.21
	U298	1.91	3.22
	H298	1.92	3.22
	G298	1.87	3.19
individual	U0	1.11	2.45
	gap	0.003 14	0.005 86

provides 3D coordinates for training data used in the dataset, but we only use molecule SMILES as inputs for model training in this work.

3 Benchmark results

3.1 Model Performance: General benchmarking

In the following, we present benchmarking results on predicting molecular targets on single-molecule datasets.

3.1.1 MoleculeNet & OGB

In the original publication of the algorithm behind Chemprop,[34] the MoleculeNet datasets were used as benchmark to compare against other non-deep-learning algorithms, such as Morgan Fingerprints used with random forest regression.[34] In this work, we do not fully repeat the original coverage of the MoleculeNet datasets. We revisit three of the datasets that continue to be of interest: QM9, HIV, and PCBA. The most significant differences in the benchmark models presented here and those presented in Ref. 34 are the improved

Table S8: Test set results for HIV and PCBA classification tasks compared with MoleculeNet (MolNet) and OGB leaderboards (higher = better). For the PCBA random split, we also report the performance with missing targets set to None (in brackets). For the PCBA scaffold split, the test set only had a single class for 'PCBA-493208' task, and therefore 'PCBA-493208' was omitted from the training, validation and test set.

Dataset	Metric	Split type	MolNet best	MolNet average	OGB best	OGB average	This work
HIV	ROC-AUC	Scaffold	0.841	0.788	0.8420	0.7936	0.8028
PCBA	PRC-AUC	Random	0.136	0.119	-	-	0.2089 (0.3840)
PCBA	AP	Random	-	-	-	-	0.2143 (0.3904)
PCBA	PRC-AUC	Scaffold	-	-	-	-	0.3012
PCBA	AP	Scaffold	-	-	0.3167	0.2716	0.3056

hyperparameter search and the adoption of the "norm" aggregation setting. For PCBA, we additionally test the performance of the model on the scaffold split that is obtained from OGB.[11]

First, we trained a multitask model on all 12 targets in the QM9 dataset, which produced an average MAE of 2.14 and RMSE of 3.96 across all targets. Though reporting averaged metrics is common, the differing orders of magnitude among the target properties biases the averaged result heavily toward targets of larger magnitudes. In Table S7, we report the test set metrics individually by task. We also trained benchmark single-task models on the U0 and HOMO-LUMO gap targets, reported in Table S7. In this benchmark, the performance observed on the single-task treatment of U0 is significantly better than the multitask version with RMSE of 2.45 and 3.21 Ha, respectively. The single-task model did not have a clear improvement on HOMO-LUMO gap performance.

The results of the benchmark Chemprop models trained on the HIV and PCBA datasets are presented in Table S8. For the PCBA dataset which has 128 classification tasks, the test scores are averaged over all tasks. The Chemprop model achieves a ROC-AUC of 0.8028 on the scaffold split for the HIV prediction. While our model underperforms compared to the best models from MoleculeNet and OGB leaderboards, our model provides better predictions than the average models from both leaderboards on the HIV scaffold split. For the PCBA random split, the Chemprop model has a PRC-AUC of 0.2089, outperforming the best model from MoleculeNet, the DeepChem graph convolutional model[35] with a PRC-AUC of 0.136.

Table S9: RMSEs for predicting logP (dimensionless) for the SAMPL6, SAMPL7, and SAMPL9 challenges.

	SAMPL6	SAMPL7	SAMPL9
Number of submissions	105	36	18
Average RMSE of submissions	1.51	1.44	2.11
Best RMSE	0.38	0.49	1.12
RMSE of this work	0.27	0.49	1.11

Compared to the best model from OGB, which uses the heterogeneous interpolation on graph[36], our model has a lower AP of 0.3028 on the PCBA scaffold split, but we are able to achieve better performance than the average models.

3.1.2 PCQM4Mv2

A benchmark model was trained on the PCQM4Mv2 dataset curated by the Open Graph Benchmark.[11] This dataset contains the molecular HOMO-LUMO gap calculated by DFT in units of eV. The benchmark Chemprop model achieved a test set MAE of 0.0956 eV and RMSE of 0.154 eV. The OGB hosts a leaderboard for performance for this dataset, based on a blinded test set. The test set used for this model was part of the open data and is expected to be similar but not the same as the test set reported on the leaderboard.

3.1.3 SAMPL

When training Chemprop to predict water-octanol partition coefficients (logP), we obtain an RMSE of 0.53 on a random test set with our conventional data splits of 80% test, 10% validation and 10% test. This corresponds to an RMSE of $0.72\,\mathrm{kcal\,mol^{-1}}$ for the transfer free energy ΔG from water to octanol at 298 K, which is related to logP by

$$\Delta G = -RT \ln 10 \cdot \log P \tag{1}$$

where R is $8.314\,\mathrm{J\,mol^{-1}\,K^{-1}}$ and T is the temperature. We then retrained a production model on the full logP dataset without any validation or test splits using the same hyperparameters to predict logP of the molecules in the SAMPL6, SAMPL7, and SAMPL9

blind prediction challenges. The performance of Chemprop is shown in Table S9, where Chemprop outperforms all other submissions from the SAMPL6, SAMPL7 and SAMPL9 challenges. We note that submissions range from quantum mechanics (QM) models and molecular mechanics (MM) models to empirical models relying on heuristic rules or machine learning, as well as mixtures thereof. Our work therefore does not only outperform other empirical models, but also a large variety of QM and MM models. In general, logP is often used in drug development, where it serves as an indicator of lipophilicity, which is known to impact the absorption, distribution, metabolism, excretion, and toxicity of drug candidates.[37] We thus demonstrate the ability of Chemprop to aid in important tasks such as drug discovery. Moreover, we note that the best performing submission in SAMPL7 was made by a biotechnology company independent of our group, using a Chemprop model trained on a different database, further highlighting the usefulness and impact of our software.

3.2 Model Performance: Specific feature demonstrations

In the following, we present benchmarking results for speciality features of Chemprop, namely the training on reactions or multiple molecules, the prediction of atom/bond-level targets or spectra, and the use of uncertainty quantification methods.

3.2.1 Atom/bond-level targets

As shown in Fig. S2, the performance of a multitask constrained D-MPNN was evaluated on a dataset containing six atomic and bond QM descriptors, with testing errors agreeing well with previous findings.[9]

BDE prediction was also examined using a single-task model for BDE and a multitask model for both BDE and partial charge. The single-task model achieved an MAE of $0.60\,\mathrm{kcal\,mol^{-1}}$, which is comparable to the testing error of 0.58 reported by the GNN model in ALFABET.[18] However, the GNN model in ALFABET was exclusively engineered for the purpose of BDE prediction, whereas the multitask model in Chemprop is capable of training

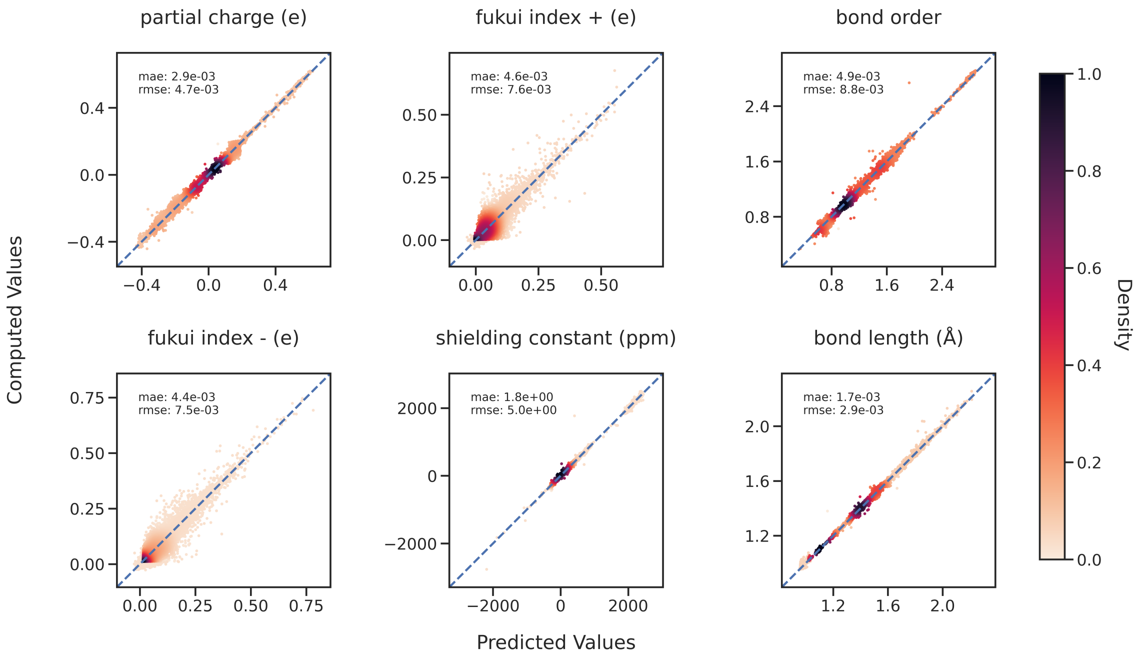

Figure S2: Comparing QM computed descriptors with multitask constrained model predictions on a held-out testing set.

on diverse atom- and bond-level properties concurrently. The multitask model, trained on both atom-level (i.e., partial charges) and bond-level (i.e., BDE) descriptors, achieved better performance with a testing MAE of 0.56 $\mathrm{kcal\,mol^{-1}}$ on BDE predictions, outperforming both the GNN model and the single-task model. This was due to the synergistic effect of co-training.

The Chemprop prediction of DDEC partial charge on small organic molecules gave a low MAE (RMSE) of 0.0053 (0.0079) e and 0.0047 (0.0097) e for dielectric constants of $\epsilon = 4$ (in protein) and $\epsilon = 78$ (in solvent), respectively. Furthermore, the models were tested on external test sets to assess their transferability to real-world applications. The MAE (RMSE) for external test set 1 (organic liquids) and external test set 2 (FDA-approved drugs) was 0.0083 (0.013) e and 0.0065 (0.012) e, respectively. The results indicated that Chemprop significantly outperformed random forest regression model, with an RMSE of 0.023 e and 0.018 e for the two external test sets, as reported by Bleiziffer et al.[19]

These findings suggest that Chemprop is promising for predicting various atom- and

Table S10: MAEs for predicting the barrier heights of organic reactions in kcal mol^{-1} for this work (top), other graph-convolutional approaches (middle, taken from Ref. 22 and 33), and simple machine learning approaches (bottom, taken from Ref. 22, 23 and 33).

	S_N2	E2	Cycloadd	RDB7	RGD1
This work	2.53	2.65	2.58	4.05	5.85
WL GNN[22]	8.49	8.04	2.76	-	-
WL ml-QM-GNN[22,33]	2.76	2.65	2.64	-	-
Chemprop reactant[22]	2.85	2.75	-	-	-
Regression QM desc.[22,33]	6.43	6.07	5.94	-	-
KRR + BoB[23]	3.49	3.27	-	-	-
KRR + SLATM[23]	2.92	2.92	-	-	-
KRR + FCHL19[23]	2.87	2.75	-	-	-
KRR + one-hot[23]	2.14	2.40	-	-	-
KNN + Morgan[33]	-	-	4.33	-	-
RF + QM desc.[33]	-	-	3.73	-	-
RF + Morgan[33]	-	-	3.50	-	-
XGBoost + QM desc.[33]	-	-	3.78	-	-
XGBoost + Morgan[33]	-	-	3.84	-	-

bond-level properties of molecules, with potential applications in drug discovery and materials science.

3.2.2 Reaction barrier heights

Table S10 summarizes the MAEs obtained for different reaction barrier height datasets.

For E2 and S_N2 reactions we can directly compare or work against the models by Stuyver et al.[22] and Heinen et al.[23] We find that Chemprop significantly outperforms the Weisfeiler-Lehman (WL) architecture from Stuyver et al.[22,33] even when adding quantum-mechanical (QM) descriptors (termed "ml-QM-GNN" in Table S10) to the WL network. We furthermore note that Ref. 22 also reports the performance of a Chemprop model, but trained it only on the reactants, not the full reactions. Here, we can directly observe the advantage offered by using the full reaction to construct the input graph representations. Chemprop furthermore outperforms the multivariate regression of quantum-mechanical descriptors of Ref. 22. Compared to the kernel ridge regression (KRR) models of Ref. 23, Chemprop outperforms the models based on BoB, SLATM, and FCHL19 representations by a large margin. The KRR model on a simple one-hot encoding of the nucleophile, electrophile and substituents close to the reactive center offers a slight performance benefit at the disadvantage of not being able

to generalize at all to new reactants or reactions.

For [3 + 2] dipolar cycloadditions, we compare Chemprop in reaction mode to WL type models with and without QM features, regressions on QM descriptors, as well as different machine learning model (k-nearest neighbor (KNN), random forests (RF) and XGBoost) on either QM descriptors or Morgan fingerprints, and find that Chemprop outperforms all other models.

A large benefit of Chemprop in reaction mode over all other architectures in Table S10 is furthermore its generality and versatility. It is straightforward to train any machine learning model on a single type of reactions (like S_N2, E2, or cycloadditions), but finding a representation and architecture that can predict reaction properties of a large variety of reactions is much more difficult. Here, we showcase the ability of Chemprop to learn from diverse reaction datasets using the RDB7[25] and the RGD1-CNHO[26] datasets. We find larger MAEs compared to the simpler single-type datasets. Albeit not reaching chemical accuracy, our models still produce state-of-the-art performances given the diversity of reactions and range of barrier heights in both datasets. In Ref. 38, a refined Chemprop model with customized atom features and pretrained on DFT data of lower level of theory yields an MAE of 2.6 $\mathrm{kcal\,mol^{-1}}$. Importantly, Chemprop does not make use of the three-dimensional structure of the reactants, products and transition states but estimates barrier heights solely from the change in bonds, thus requiring minimal information to predict a new reaction. We furthermore note that simpler approaches such as models trained only on reactant structures or descriptors are not applicable to diverse reaction datasets.

3.2.3 UV/Vis absorption

Chemprop achieved an MAE of 15.5 nm, RMSE of 29.7 nm, and R^2 of 0.920 on our dataset of experimental absorption peak wavelengths across a diverse set of dye molecules in a variety of solvents. This was previously demonstrated to outperform state-of-the-art fingerprint-based methods[39]. Our train-validation-test splitting for this task was constrained such that all

Table S11: Uncertainty evaluation metrics (dimensionless) for QM9 gap predictions. *NLL*: negative log likelihood; ρ: Spearman rank correlation; *ENCE*: expected normalized calibration error; *MA*: miscalibration area. The arrows indicate if smaller or larger values indicate better performance.

Method	NLL ($\downarrow$)	ρ ($\uparrow$)	ENCE ($\downarrow$)	MA ($\downarrow$)
Ensemble	-3.94	0.33	0.21	0.14
Evidential	-4.06	0.47	0.27	0.15
MVE	-4.13	0.47	0.17	0.10

measurements of the same molecule in different solvents would be assigned to the same split to avoid data leakage. Previous work has shown that data leakage can lead to overly-optimistic estimates of generalization ability on similar datasets.[39] Any work on multi-molecule tasks should carefully consider the implications of the choice of splitting technique to avoid data leakage from highly correlated samples (in cases such as measurements of the same property in different solvents) or from duplicated samples with flipped molecule columns (in cases of symmetric multi-molecule properties).

3.2.4 IR spectra

Chemprop spectra prediction was benchmarked using gas-phase IR absorbance data provided publicly by NIST.[31] Similarity between the predicted spectra and the target spectra are assessed using Spectral Information Divergence, SID. The benchmark average SID for predictions on the test set was 0.27. Qualitatively, a SID value of 0.27 is a good prediction which generally tends to match the location and magnitude of all major peaks and the location of most minor peaks, while smoothing some of the details of peak shape. To give some context to this value, we also provide some simple baselines for comparison. The average SID of a uniform distribution against the dataset was 2.52. The average SID of a roundrobin pairing of every spectrum in the dataset with every other spectrum in the dataset was 2.89. The average SID of a normalized sum of all the spectra against each individual member of the dataset was 1.45.

3.2.5 Uncertainty estimation for QM9 gap

Table S11 summarizes the performance of three uncertainty quantification (UQ) methods (ensemble, evidential, and mean-variance estimation (MVE)) selected from Chemprop's available UQ options. For the evidential uncertainty, we used the total uncertainty (the sum of aleatoric and epistemic components). We trained all models on the gap values from the QM9 dataset and calibrated the predictions using the z-scaling method[40] with the standard deviation as the regression calibrator metric. We then evaluated the methods based on four metrics: negative log likelihood (NLL), Spearman rank correlation (ρ), expected normalized calibration error (ENCE), and miscalibration area (MA). On this task, we observe that MVE performs the best across all four metrics, while ensemble performs the worst across all metrics, with evidential in between. However, we emphasize that UQ performance can vary depending on task, dataset size, representation, and other factors. The results presented here simply serve as an illustration of Chemprop UQ capabilities; we refer readers to substantial UQ benchmarking work done previously for further discussion of the merits and pitfalls of various methods and metrics on chemical and materials data.[41–46]

3.3 Timing

Training and inference timing benchmarks for Chemprop can be found in Tables S12, S13, and S14. These benchmarks were measured on three systems: a compute cluster node with CPU only, a compute cluster node with a GPU resource, and a laptop. We used an Intel Xeon Platinum 8260 processor (2.4 GHz, 48 CPU cores) for cluster CPU benchmarks and an Intel Xeon Gold 6248 (2.5 GHz, 40 CPU cores) processor with an Nvidia Volta V100 GPU for the cluster GPU benchmark timing. Both devices are part of the MIT Supercloud.[47] For both systems, we restricted the maximum numbers of CPU cores accessible to Chemprop to 8. For laptop timing, we used a Thinkpad X1 Carbon with an Intel Core i7-1280P (1.8 GHz, 14 CPU cores) processor and no enabled GPU. Our benchmark datasets were randomly

Table S12: Train times in hours:minutes:seconds for subsets of the QM9 dataset

Device	1k	10k	100k
Laptop	0:01:53	0:19:15	3:24:57
Cluster CPU	0:00:46	0:07:06	1:15:18
Cluster GPU	0:00:19	0:02:48	0:31:06

sampled subsets of the QM9 HOMO-LUMO gap targets with sizes of 100,000, 10,000, and 1,000.

The training times for Chemprop models found in Table S12 includes all training processes, including time for data preprocessing and model evaluation. This training was carried out with a 80/10/10 training-validation-test split of the data. The hyperparameters were chosen to be in the typical range used for datasets of this size: hidden size of 1000, feed forward hidden size of 1000, 4 message passing layers, 2 feed forward layers, and 50 epochs. The training times found in Table S13 are average training times on a per epoch basis. This average time includes the feed forward, loss function calculation, model update, and model evaluation steps. The average time excludes the time taken in the first training epoch, data preprocessing, and final model evaluation. Inference timing benchmarks can be found in Table S14. These times include all inference processes, including postprocessing of the predictions.

Training time shows significant speed improvement when moving from the laptop platform to the cluster CPU system and further improvement moving from the cluster CPU system to the cluster GPU system. This trend is followed across the tested dataset sizes. In each case, the speedup is greater than a factor of 2. Training for single models on moderately sized datasets can be carried out reasonably even on a laptop. For large datasets, hyperparameter optimization, and model structures involving many submodels, training on cluster resources or using a GPU is recommended. Inference times in the 10,000 and 100,000 dataset sizes are also improved when moving from laptop to cluster CPU to cluster GPU, but the progressive improvement is smaller than for training. Inference using any of the system levels tested is relatively fast for these dataset sizes.

Table S13: Average training times for an epoch in seconds for subsets of the QM9 dataset, excluding the first epoch.

Device	1k	10k	100k
Laptop	2.2	23.1	245
Cluster CPU	0.9	8.4	90
Cluster GPU	0.3	3.2	36

Table S14: Inference times in hours:minutes:seconds for subsets of the QM9 dataset.

Device	1k	10k	100k
Laptop	0:00:01	0:00:11	0:01:34
Cluster CPU	0:00:02	0:00:08	0:01:12
Cluster GPU	0:00:03	0:00:07	0:00:46

References

(1) Delaney, J. S. ESOL: Estimating Aqueous Solubility Directly from Molecular Structure. *J. Chem. Inf. Comput.* **2004**, *44*, 1000–1005.

(2) Chemprop: Molecular Property Prediction. `https://www.github.com/chemprop/chemprop`, (accessed April 6 2023).

(3) Chemprop. `https://chemprop.readthedocs.io/en/latest/`, (accessed April 6 2023).

(4) Hinton, G. E.; Srivastava, N.; Krizhevsky, A.; Sutskever, I.; Salakhutdinov, R. R. Improving Neural Networks by Preventing Co-adaptation of Feature Detectors. *arXiv Preprint* **2012**, arXiv:1207.0580.

(5) Chung, Y.; Green, W. H. Machine Learning from Quantum Chemistry to Predict Experimental Solvent Effects on Reaction Rates. *ChemRxiv Preprint* **2023**, ChemRxiv.

(6) Bergstra, J.; Bardenet, R.; Bengio, Y.; Kégl, B. Algorithms for Hyper-Parameter Optimization. *Adv. Neural Inf. Process. Syst.* **2011**, *24*.

(7) Bergstra, J.; Yamins, D.; Cox, D. Making a Science of Model Search: Hyperparameter

Optimization in Hundreds of Dimensions for Vision Architectures. *Proceedings of the International Conference on Machine Learning* **2013**, 115–123.

(8) Landrum, G. RDKit: Open-Source Cheminformatics. 2006; https://www.rdkit.org/.

(9) Guan, Y.; Coley, C. W.; Wu, H.; Ranasinghe, D.; Heid, E.; Struble, T. J.; Pattanaik, L.; Green, W. H.; Jensen, K. F. Regio-Selectivity Prediction with a Machine-Learned Reaction Representation and On-the-Fly Quantum Mechanical Descriptors. *Chem. Sci.* **2021**, *12*, 2198–2208.

(10) Wu, Z.; Ramsundar, B.; Feinberg, E. N.; Gomes, J.; Geniesse, C.; Pappu, A. S.; Leswing, K.; Pande, V. MoleculeNet: A Benchmark for Molecular Machine Learning. *Chem. Sci.* **2018**, *9*, 513–530.

(11) Hu, W.; Fey, M.; Zitnik, M.; Dong, Y.; Ren, H.; Liu, B.; Catasta, M.; Leskovec, J. Open Graph Benchmark: Datasets for Machine Learning on Graphs. 2021.

(12) Filtering Chemical Libraries. https://practicalcheminformatics.blogspot.com/2018/08/filtering-chemical-libraries.html, (accessed November 14 2023).

(13) Wang, Y.; Xiao, J.; Suzek, T. O.; Zhang, J.; Wang, J.; Zhou, Z.; Han, L.; Karapetyan, K.; Dracheva, S.; Shoemaker, B. A.; Bolton, E.; Gindulyte, A.; Bryant, S. H. PubChem's BioAssay Database. *Nucleic Acids Res.* **2012**, *40*, D400–D412.

(14) Ramakrishnan, R.; Dral, P. O.; Rupp, M.; Von Lilienfeld, O. A. Quantum Chemistry Structures and Properties of 134 kilo Molecules. *Sci. Dat.* **2014**, *1*, 140022.

(15) The SAMPL Challenges. https://github.com/samplchallenges, (accessed March 15, 2023).

(16) Koscher, B.; Canty, R. B.; McDonald, M. A.; Greenman, K. P.; McGill, C. J.; Bilodeau, C. L.; Jin, W.; Wu, H.; Vermeire, F. H.; Jin, B., et al. Autonomous, Multi-

Property-Driven Molecular Discovery: From Predictions to Measurements and Back. *Science* **2023**, eadi1407.

(17) St. John, P. C.; Guan, Y.; Kim, Y.; Kim, S.; Paton, R. S. BDE-db: A Collection of 290,664 Homolytic Bond Dissociation Enthalpies for Small Organic Molecules. `https://doi.org/10.6084/m9.figshare.10248932.v1`, 2019; (accessed March 22, 2023).

(18) St. John, P. C.; Guan, Y.; Kim, Y.; Kim, S.; Paton, R. S. Prediction of Organic Homolytic Bond Dissociation Enthalpies at Near Chemical Accuracy with Sub-Second Computational Cost. *Nat. Commun.* **2020**, *11*, 2328.

(19) Bleiziffer, P.; Schaller, K.; Riniker, S. Machine Learning of Partial Charges Derived from High-Quality Quantum-Mechanical Calculations. *J. Chem. Inf. Model.* **2018**, *58*, 579–590.

(20) von Rudorff, G. F.; Heinen, S. N.; Bragato, M.; von Lilienfeld, O. A. Thousands of Reactants and Transition States for Competing E2 and S2 Reactions. *Mach. Learn.: Sci. Technol.* **2020**, *1*, 045026.

(21) Heid, E.; Green, W. H. Machine Learning of Reaction Properties via Learned Representations of the Condensed Graph of Reaction. *J. Chem. Inf. Model.* **2022**, *62*, 2101–2110.

(22) Stuyver, T.; Coley, C. W. Quantum Chemistry-Augmented Neural Networks for Reactivity Prediction: Performance, Generalizability, and Explainability. *J. Chem. Phys.* **2022**, *156*, 084104.

(23) Heinen, S.; von Rudorff, G. F.; von Lilienfeld, O. A. Toward the Design of Chemical Reactions: Machine Learning Barriers of Competing Mechanisms in Reactant Space. *J. Chem. Phys.* **2021**, *155*, 064105.

(24) Stuyver, T.; Jorner, K.; Coley, C. W. Reaction Profiles for Quantum Chemistry-Computed [3+ 2] Cycloaddition Reactions. *Sci. Dat.* **2023**, *10*, 66.

(25) Spiekermann, K.; Pattanaik, L.; Green, W. H. High Accuracy Barrier Heights, Enthalpies, and Rate Coefficients for Chemical Reactions. *Sci. Dat.* **2022**, *9*, 417.

(26) Zhao, Q.; Vaddadi, S. M.; Woulfe, M.; Ogunfowora, L. A.; Garimella, S. S.; Isayev, O.; Savoie, B. M. Comprehensive Exploration of Graphically Defined Reaction Spaces. *Sci. Dat.* **2023**, *10*, 145.

(27) Joung, J. F.; Han, M.; Jeong, M.; Park, S. Experimental Database of Optical Properties of Organic Compounds. *Sci. Dat.* **2020**, *7*, 1–6.

(28) Ju, C.-W.; Bai, H.; Li, B.; Liu, R. Machine Learning Enables Highly Accurate Predictions of Photophysical Properties of Organic Fluorescent Materials: Emission Wavelengths and Quantum Yields. *J. Chem. Inf. Model.* **2021**, *61*, 1053–1065.

(29) Venkatraman, V.; Raju, R.; Oikonomopoulos, S. P.; Alsberg, B. K. The Dye-Sensitized Solar Cell Database. *J. Cheminf.* **2018**, *10*, 1–9.

(30) Venkatraman, V.; Kallidanthiyil Chellappan, L. An Open Access Data Set Highlighting Aggregation of Dyes on Metal Oxides. *Data* **2020**, *5*, 45.

(31) NIST Mass Spectrometry Data Center, In *"Infrared Spectra" in NIST Chemistry WebBook, NIST Standard Reference Database Number 69*; Lindstrom, P. J., Mallard, W. G., Eds.; National Institute of Standards and Testing: Gaithersburg, MD, `http://webbook.nist.gov` (Accessed 2019-10-3).

(32) Nakata, M.; Shimazaki, T. PubChemQC Project: A Large-Scale First-Principles Electronic Structure Database for Data-Driven Chemistry. *J. Chem. Inf. Model.* **2017**, *57*, 1300–1308.

(33) Stuyver, T.; Coley, C. Machine Learning-Guided Computational Screening of New Candidate Reactions with High Bioorthogonal Click Potential. *Chem. Eur. J.* **2023**, e202300387.

(34) Yang, K.; Swanson, K.; Jin, W.; Coley, C.; Eiden, P.; Gao, H.; Guzman-Perez, A.; Hopper, T.; Kelley, B.; Mathea, M.; Palmer, A.; Settels, V.; Jaakkola, T.; Jensen, K.; Barzilay, R. Analyzing Learned Molecular Representations for Property Prediction. *J. Chem. Inf. Model.* **2019**, *59*, 3370–3388.

(35) Altae-Tran, H.; Ramsundar, B.; Pappu, A. S.; Pande, V. S. Low Data Drug Discovery with One-shot Learning. *arXiv Preprint* **2016**, arXiv:1611.03199.

(36) Heterogeneous Interpolation on Graph. `https://github.com/TencentYoutuResearch/HIG-GraphClassification`, 2021; (accessed May 28 2023).

(37) Sun, Y.; Hou, T.; He, X.; Man, V. H.; Wang, J. Development and Test of Highly Accurate Endpoint Free Energy Methods. 2: Prediction of Logarithm of n-Octanol–Water Partition Coefficient (logP) for Druglike Molecules using MM-PBSA Method. *J. Comp. Chem.* **2023**, *44*, 1300–1311.

(38) Spiekermann, K. A.; Pattanaik, L.; Green, W. H. Fast Predictions of Reaction Barrier Heights: Toward Coupled-Cluster Accuracy. *J. Phys. Chem. A* **2022**, *126*, 3976–3986.

(39) Greenman, K. P.; Green, W. H.; Gómez-Bombarelli, R. Multi-Fidelity Prediction of Molecular Optical Peaks with Deep Learning. *Chem. Sci.* **2022**, *13*, 1152–1162.

(40) Levi, D.; Gispan, L.; Giladi, N.; Fetaya, E. Evaluating and calibrating uncertainty prediction in regression tasks. *Sensors* **2022**, *22*, 5540.

(41) Scalia, G.; Grambow, C. A.; Pernici, B.; Li, Y.-P.; Green, W. H. Evaluating Scalable Uncertainty Estimation Methods for Deep Learning-Based Molecular Property Prediction. *J. Chem. Inf. Model.* **2020**, *60*, 2697–2717.

(42) Tran, K.; Neiswanger, W.; Yoon, J.; Zhang, Q.; Xing, E.; Ulissi, Z. W. Methods for Comparing Uncertainty Quantifications for Material Property Predictions. *Machine Learning: Science and Technology* **2020**, *1*, 025006.

(43) Hirschfeld, L.; Swanson, K.; Yang, K.; Barzilay, R.; Coley, C. W. Uncertainty Quantification using Neural Networks for Molecular Property Prediction. *J. Chem. Inf. Model.* **2020**, *60*, 3770–3780.

(44) Nigam, A.; Pollice, R.; Hurley, M. F.; Hickman, R. J.; Aldeghi, M.; Yoshikawa, N.; Chithrananda, S.; Voelz, V. A.; Aspuru-Guzik, A. Assigning Confidence to Molecular Property Prediction. *Expert Opin. Drug Discov.* **2021**, *16*, 1009–1023.

(45) Soleimany, A. P.; Amini, A.; Goldman, S.; Rus, D.; Bhatia, S. N.; Coley, C. W. Evidential Deep Learning for Guided Molecular Property Prediction and Discovery. *ACS Cent. Sci.* **2021**, *7*, 1356–1367.

(46) Heid, E.; McGill, C. J.; Vermeire, F. H.; Green, W. H. Characterizing Uncertainty in Machine Learning for Chemistry. *J. Chem. Inf. Model.* **2023**, *63*, 4012–4029.

(47) Reuther, A.; Kepner, J.; Byun, C.; Samsi, S.; Arcand, W.; Bestor, D.; Bergeron, B.; Gadepally, V.; Houle, M.; Hubbell, M., et al. Interactive Supercomputing on 40,000 Cores for Machine Learning and Data Analysis. *Proceedings of the IEEE High Performance Extreme Computing Conference* **2018**, 1–6.
